# Handgrip Strength in Children and Adolescents Aged 3 to 16 Years and Residing in Spain: New Reference Values

**DOI:** 10.3390/children12040471

**Published:** 2025-04-06

**Authors:** F. Zárate-Osuna, A. G. Zapico, M. González-Gross

**Affiliations:** 1 ImFINE Research Group, Department of Health and Human Performance, Facultad de Ciencias de la Actividad Física y del Deporte-INEF, Universidad Politécnica de Madrid, 28040 Madrid, Spain; a.gzapico@upm.es (A.G.Z.); marcela.gonzalez.gross@upm.es (M.G.-G.); 2 Pediatric Department, Quirónsalud Sur Hospital, 28922 Alcorcón, Spain; 3 Pediatric Department, Quirónsalud Toledo Hospital, 45001 Toledo, Spain; 4 Centro de Investigación Biomédica en Red Fisiopatología de la Obesidad y la Nutrición (CIBEROBN), Institute of Health Carlos III, 28029 Madrid, Spain

**Keywords:** dynamometry, strength, physical condition, body composition

## Abstract

**Introduction:** Handgrip strength, measured by dynamometry (HGD), is a key measure in assessing physical condition and nutritional status. Its correlation with anthropometric measures and body composition makes it an accessible method for the evaluation of cardiovascular health. This study aimed to develop a new reference for right-hand dynamometry in the Spanish population and compare it with previous references. **Material and Methods:** A total of 3281 subjects aged 3 to 16 years (1608 females) from the PESCA, PASOS, and ASOMAD projects were included. Handgrip strength was measured using the same methodology in all cases. Data on age, weight, height, and BMI were collected, and the handgrip strength per kilogram of body weight was calculated. Sexual dimorphism in the temporal development of strength was analyzed, and multiple correlations were established between dynamometry and anthropometric variables. **Results:** Percentile curves and tables for dynamometry are presented for each sex, including data from as early as 3 years old, for the first time. **Conclusions**: Sexual dimorphism in strength development is confirmed, becoming more pronounced with puberty. In absolute terms, our study shows a decrease in handgrip strength among adolescents, occurring earlier and more markedly in females. When compared to the previous literature, the 16-year-old male adolescents in our study exhibited lower strength than those from 40 and 16 years ago.

## 1. Introduction

Handgrip dynamometry (HGD) is a tool used for the diagnosis and assessment of health in various populations, including pediatric and adolescent age groups [1]. This technique measures the maximal static muscle strength through the motor function of the finger flexor muscles, as well as the muscles of the hypothenar and thenar regions and the intrinsic muscles of the hand [2]. This measurement provides a reliable estimate of an individual’s physical fitness and nutritional status [3]. Handgrip strength, assessed through dynamometry, varies with age and is influenced by different health or disease conditions throughout life. The accessibility and reproducibility of this test make it particularly relevant for both the clinical assessment of development in children and adolescents and the monitoring of physical performance during these stages. Its relationship with body mass index (BMI) and the evidence supporting its correlation with body composition (lean mass, fat-free mass, and bone mineral content) [4] underscore its importance as a cardiovascular health marker. Furthermore, several studies have reported associations between low handgrip strength and increased cardiometabolic risk factors in pediatric populations, reinforcing its potential as a marker of cardiovascular health from early ages [5,6].

Given its utility in assessing physical fitness and nutritional status, muscle performance, and/or sports performance, as well as in the overall evaluation of cardiovascular health, it is crucial to understand the evolution of strength measured by dynamometry throughout childhood and adolescence to establish a reference pattern. It is well known that sex-related differences exist in the development of strength measured by dynamometry over time [7]. These differences are reflected not only in the absolute strength values at each stage of development but also in the rate of variation and the chronological timing at which peak values are reached. Thus, establishing a reference pattern for handgrip dynamometry, similarly to the assessment of growth in weight, height, and BMI using percentile charts and curves in child health programs, would allow us to (A) position each child or adolescent in comparison with the normative reference, thereby obtaining a snapshot of their current status; (B) evaluate their progression over time and determine whether their development is appropriate in terms of health; (C) assess whether variations in the rate of strength acquisition follow a physiological pattern or are related to specific conditions such as obesity or athletic training; and (D) compare individuals and populations.

Among the Spanish references published to date, Marrodán Serrano et al. (2009) presented tables with data collected since 2006 from healthy Spanish children and adolescents, proposed as reference values [3]. This study emphasized the relationship between handgrip strength and body composition, particularly lean mass. Since the data collection for this reference, 18 years have passed, during which significant changes in lifestyle habits have been observed, including the abandonment of the Mediterranean diet, increased sedentary behavior, and decreased physical activity. These changes have contributed to an unfavorable health scenario, with alarming prevalence rates of overweight and obesity among children and adolescents [8]. Furthermore, in March 2020, the COVID-19 pandemic had a significant impact on global health, affecting, among other factors, the physical activity and dietary habits of children and adolescents worldwide [9,10,11]. These lifestyle shifts may help to explain the decline in handgrip strength observed globally over recent decades, as shown in a systematic analysis of more than 2 million children and adolescents from 1967 to 2017 [12].

The primary objective of this study is to provide an updated reference for handgrip dynamometry in the Spanish pediatric population. The sample used for this analysis, initially comprising 3400 children and adolescents aged 3 to 17 years, comes from three child health research initiatives in Spain. These initiatives include, within their respective protocols, the measurement of handgrip dynamometry using the same standardized methodology across all three studies: (1) the *Programa Escolar de Salud Cardiovascular* (PESCA), a program for the prevention, diagnosis, and management of childhood overweight and obesity in Spain, primarily aimed at reducing the prevalence of overweight since 2018 [13]; (2) the PASOS study, conducted by the GASOL Foundation, which assesses lifestyle and health indicators in Spanish children and adolescents aged 8 to 16 years and their families [14]; and (3) the ASOMAD study, led by the ImFINE Research Group, which analyzes physical activity, sedentary behavior, and obesity in children from the city of Madrid [15]. A secondary objective is to provide a detailed description of the normal variation in handgrip strength according to age and sex for this new reference. Finally, we aim to analyze and compare the trends observed in our reference with previously established references for the Spanish population. In this context, the updated reference values provided in this study may be used by pediatricians and primary care professionals as a complementary tool to assess muscular fitness and cardiovascular health in clinical or public health settings.

In summary, this study seeks to determine whether the current handgrip strength levels in the Spanish child and adolescent population differ from previous national references and to provide updated normative values stratified by age and sex.

## 2. Materials and Methods

### 2.1. Study Desing

This was a cross-sectional, multicenter, population-based observational study. The data were collected from three pediatric health research initiatives conducted in Spain between 2018 and 2022: the PESCA program, the PASOS study, and the ASOMAD study. 

### 2.2. Subjects and Data Gathering

The final sample consisted of 3281 children and adolescents (1608 females and 1673 males) aged between 3 and 16.97 years. The sample was drawn from three health programs and/or epidemiological studies. In all three studies, participation was voluntary and required informed consent from the participants’ parents or legal guardians. The PESCA program, which conducts annual data collection, contributed 1772 participants across its first four waves, from November 2018 to June 2022. The PASOS study provided 777 participants, with data collection occurring in two waves between March 2019 and June 2022. The ASOMAD study contributed 851 participants, with data collection also carried out in two waves between October 2020 and April 2021. All data were collected following standardized procedures applied consistently across the three studies

Of the initial 3400 students, those younger than 3 years or older than 17 years were excluded, as these subgroups were too small to allow for a sufficiently representative analysis (36 subjects). Additionally, participants with detected errors in the measurement or interpretation of dynamometry values were excluded (39 subjects). Although the PESCA protocol includes the measurement of handgrip strength in the dominant hand, both the PASOS and ASOMAD studies assessed grip strength in both hands. To ensure the comparability of the data across the three sources, PESCA participants who reported left-hand dominance and performed the test with their left hand were excluded (44 students). Thus, the data presented serve as a reference for right-hand grip strength in a sample of 3281 children and adolescents.

Each participant’s parents or legal guardians provided informed consent for their child’s participation in this study. The three protocols (PESCA, PASOS, and ASOMAD) adhered to the ethical standards of the Declaration of Helsinki and complied with data protection regulations—specifically, the General Data Protection Regulation (EU) 2016/679 and the Spanish Organic Law 3/2018 on Data Protection and Guarantee of Digital Rights. In addition to compliance with the General Data Protection Regulation (EU) 2016/679 and the Spanish Organic Law 3/2018, the PESCA protocol is embedded within a clinical care framework, which ensures the highest standards of data confidentiality. Data were pseudonymized, securely stored, and only accessible to authorized personnel.

The PESCA program’s protocol was approved by the Research Ethics Committee (CEIm-FJD) of Fundación Jiménez Díaz on 9 October 2018 (Record No. 18/18). The protocols for the PASOS and ASOMAD studies were approved by the following committees: the Research Ethics Committee for Medicinal Products (CEIm) of Fundació Sant Joan de Déu, Barcelona, on 27 January 2022 (CEIm Code: PIC-179-18); the Ethics Committee of the Technical University of Madrid (Approval No. 20200727-1), on 1 September 2020.

The vast majority of the participants (99.3%) were students from various schools in the city of Madrid and the Madrid region, including public, private, and semi-private (*concertado*) schools, according to the corresponding sampling procedures. A small proportion (0.7%; 23 students) came from a semi-private school in Toledo (Castilla-La Mancha).

### 2.3. Study Variables

From the numerous measurements included in the protocols of the three projects, the following variables were considered for this study: age (years), weight (kg), height (cm), BMI (kg/m^2^), and handgrip strength (kg). Additionally, the prevalence of overweight and obesity was included, using the International Obesity Task Force (IOTF) cutoff points as a reference [16].

The weekly screen time for non-academic purposes (in hours) and the weekly time spent on sports or physical activity (excluding physical education class hours) were recorded for 1654 and 1680 participants, respectively, within the PESCA program sample. These data were collected using the questionnaire included in the PESCA protocol.

As a derived variable, handgrip strength relative to body weight (expressed as kg HGD/kg body weight) was also included. Table 1 presents the descriptive statistics of the considered variables, stratified by sex.

### 2.4. Instruments

The following equipment was used for data collection.

Height measurement (cm): SECA stadiometer/height rod—SECA 213 (2019), SECA 206 (2018), or SECA 220 (2017)—with precision of 0.1 cm (SECA, Hamburg, Germany).
Weight measurement (kg): Tanita SC-240MA (Tanita Europe BV, Amsterdam, The Netherlands), a scientifically validated standard for both the U.S. and the EU (2018), providing weight, BMI, and body fat and water percentage data.
Handgrip dynamometry (kg): Takei Physical Fitness Test T.K.K. 5001 GRIP A analog dynamometer (2018) with a measurement range of 0 to 100 kg (Takei, Tokyo, Japan).

For handgrip strength measurement, the first trial was conducted with the participant standing, with the arm extended parallel to the torso while holding the dynamometer. At the given signal, the participant exerted the maximum grip strength for up to 10 s using the right hand while being encouraged to apply the maximum effort. After a recovery period of 1 to 2 min, the test was repeated, with the highest recorded value considered as the valid result. Although the three studies (PESCA, PASOS, ASOMAD) included additional variables of interest, the handgrip strength assessment followed an identical protocol across all of them. To ensure data comparability, only right-hand measurements were analyzed, as they were consistently available in all three datasets. Furthermore, all participants were drawn from similar geographical and socioeconomic contexts. Although formal inter-rater reliability tests were not conducted, all evaluators followed a standardized protocol and received prior training to ensure consistency in handgrip strength measurement across all study sites.

### 2.5. Statistical Analysis

The statistical analysis was performed using the SPSS software, version 25.0, for Mac (IBM). The sample was segmented into whole-year age groups for each sex (ages 3 to 16 years). The mean (M), standard deviation (SD), and percentiles 3, 10, 20, 25, 30, 40, 50, 60, 75, 80, and 90 were calculated for handgrip strength. The 97th percentile could only be calculated for females, as the distribution of male values in the upper tail was insufficient to ensure a reliable estimation. For the remaining variables, the M and SD or prevalence (%) with a 95% confidence interval were calculated, depending on the variable type.

The normality of the distribution for the main continuous variables was tested using the Kolmogorov–Smirnov test, which showed a statistically significant deviation from normality (*p* < 0.001). Therefore, non-parametric tests were applied where appropriate. The Mann–Whitney U test was used to compare the mean values of handgrip strength, age, weight, height, BMI, hours of sports participation, and screen time between sexes. Student’s *t*-test was applied to compare differences in handgrip strength relative to body weight (HGD/kg). The prevalence of overweight and obesity between sexes was compared using the chi-squared test. Statistical significance was set at 0.05 for all analyses. The Spearman’s rho correlation coefficient was used to assess the relationship between handgrip strength and anthropometric variables. Data visualization and graphical representations were conducted using Microsoft^®^ Excel for Mac (version 16.88, Microsoft 365 license).

## 3. Results

We present a new reference for handgrip dynamometry in the Spanish population aged 3 to 16 years. Percentile curves for both sexes are shown in Appendix A.

For the first time in Spanish reference studies, handgrip strength values are provided for the early childhood stages. A total of 645 subjects (19.7% of the sample) were younger than 7 years old.

**Figure 1:**
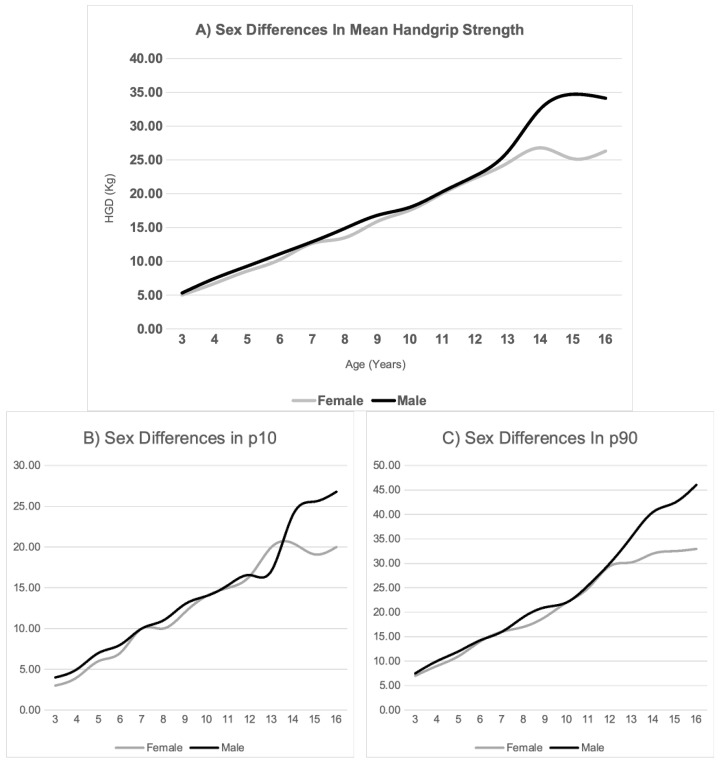
Sexual dimorphism in mean handgrip strength (**A**) at the 10th percentile (**B**) and at the 90th percentile (**C**).

**Figure 2:**
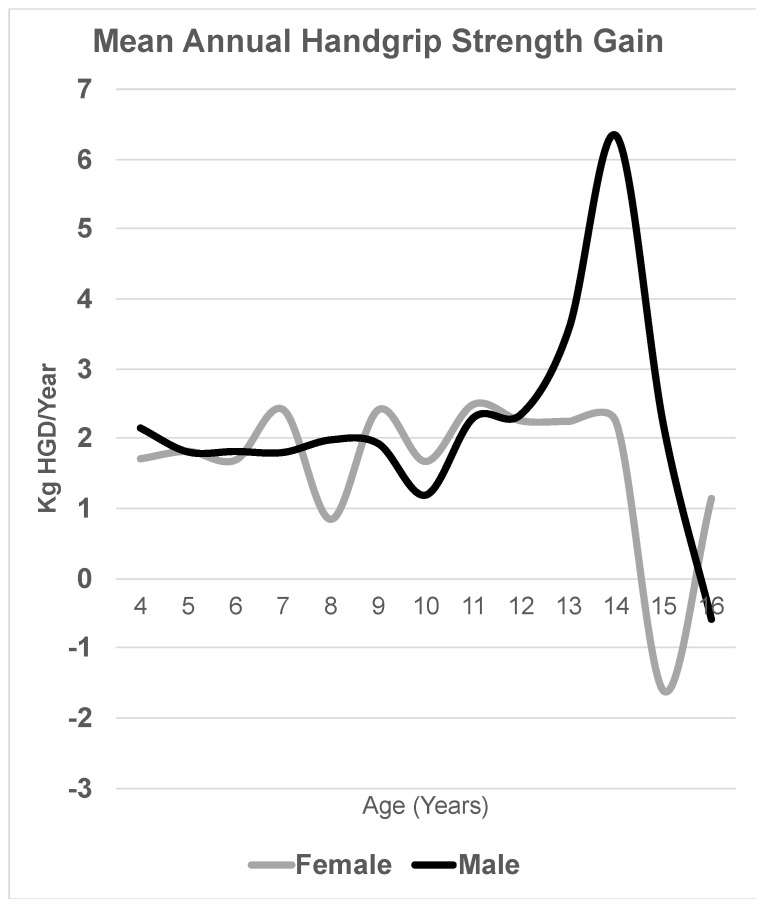
Mean annual handgrip strength gain by sex.

In this regard, as shown in Figure 1A, handgrip strength progressively increases—even in the early childhood stages—in both sexes, until the age of 12, at which point significant differences emerge in both the absolute values and the rate of variation. This trend is also reflected in the average annual strength gain (Figure 2). While males experience a peak in strength gain at 13–14 years (6.33 kg/year), the maximum increase in females is observed between 11 and 12 years (2.48 kg/year).

Table 2 presents the handgrip strength percentiles, mean, and standard deviation for the right hand, as well as the number of subjects (*n*) in each age group (in completed years). The *p*-value for sex comparisons is also provided. In this regard, although the difference in strength between sexes is visually evident in the three oldest age groups in our sample (14, 15, and 16 years), it is noteworthy that the mean strength—always higher in absolute terms in males—is statistically significant, according to the Mann–Whitney test, even in the younger age groups of 4 and 5 years and 8 and 9 years.

The results indicate the presence of pronounced sexual dimorphism in the development of muscle strength during childhood and adolescence, which becomes more evident during puberty. This pattern is characterized by an earlier but less pronounced increase in females, whereas, in males, strength development occurs later but is more substantial.

A correlation analysis between handgrip strength and anthropometric values has been included (Table 3). In this regard, our data confirm the strong relationship between handgrip strength and the subject’s weight, height, and age. The Spearman’s rho correlation coefficients showed strong positive associations between handgrip strength and anthropometric variables, particularly age (ρ = 0.87), height (ρ = 0.89), and weight (ρ = 0.86), all statistically significant (*p* < 0.001).

## 4. Discussion

Handgrip dynamometry is a key tool in assessing physical fitness in the field of exercise and sports sciences. Additionally, its simplicity in measurement and interpretation makes it an accessible and easily implementable method in routine pediatric consultations from an early age. The importance of early assessment and diagnosis lies in its ability to prevent and manage cardiovascular diseases in children and adolescents [17]. Our data support the application of this tool from the age of 3, a stage in which the connection with the Child Health Program remains strong, allowing for systematic follow-up.

Handgrip strength is closely associated with anthropometric indicators such as weight, height, and muscle mass, making its measurement a reliable reflection of a child’s health and nutritional status [18,19,20]. The development of specific curves and percentiles, as presented in this study, represents a crucial update to existing references, incorporating even the impact of the COVID-19 pandemic period, for use in pediatric consultations. We believe that its analysis and interpretation within the clinical context of each child or adolescent enhance the diagnostic capacity available for the assessment of pediatric health. Similarly to the ways in which growth and BMI charts allow for an initial diagnosis of short stature or overweight [21], the use of handgrip strength percentiles can help to identify children at risk of developing issues related to growth, nutritional status, or muscle health [18,19].

In this regard, we propose the incorporation of handgrip dynamometry into general pediatric consultations, particularly during routine health check-ups. This tool would not only allow for an accurate assessment of strength and an approximation of the child’s body composition but also contribute to a broader preventive approach. Its strong association with lean mass, overall physical fitness, and nutritional status [7,22] positions handgrip dynamometry as a reliable and practical indicator, capable of detecting imbalances in muscle development and significantly contributing to the promotion of overall health in childhood and adolescence [18,19]. It is also important to distinguish between physical activity and sedentary behavior, as both are independent factors influencing children’s health. While regular physical activity promotes muscular development and overall fitness, excessive sedentary time has been linked to poorer muscle strength and cardiometabolic outcomes, regardless of activity levels [23].

As previously mentioned, the new reference presented in this study corresponds to the right hand. From a methodological standpoint, this approach allows for the integration of data from three independent sources (PESCA, PASOS, and ASOMAD) without compromising comparability. It is widely recognized that the right hand tends to exhibit greater grip strength in most individuals, regardless of hand dominance, with these differences being more pronounced in right-handed individuals and less significant in left-handed individuals [24,25]. Therefore, the reference that we propose can be considered representative of the maximal handgrip strength. However, as left-handed individuals were excluded from the PESCA sample, future studies should consider evaluating both hands to assess potential differences in strength patterns across handedness.

In addition to the progressive increase in handgrip strength from the early stages and the pronounced sexual dimorphism observed from puberty onwards, our results provide an interesting perspective when analyzing the extreme percentiles (p10 and p90), which represent the weakest and strongest children in terms of strength. As shown in Figure 1B,C, the pattern of sex differences varies depending on the percentile. At p10, the strength difference favoring males becomes visually significant from the age of 14. In contrast, at p90, this difference follows a distinct linear pattern in favor of males starting from age 12.

This pattern not only reflects a greater strength gain in the higher percentiles but also indicates greater variability in the distribution of strength within the male group compared to the female group. Notably, at p10, girls surpass boys in strength at age 13, possibly due to the earlier onset of pubertal changes in females compared to males. In addition to pubertal timing, this difference in strength gain may also be influenced by sex-related differences in hormonal responses, lean mass development, and the levels of physical activity during adolescence.

The results obtained in this study provide a unique opportunity to analyze the evolution of handgrip strength in the Spanish pediatric population in comparison with historical references. This comparative analysis is essential in understanding potential changes in muscle development over time and in evaluating factors that may have influenced these differences, such as lifestyle trends or recent events like the COVID-19 pandemic. Although the present study was not designed to isolate the specific impact of the pandemic, its potential influence on physical development should be investigated in future research. In this context, we examined the similarities and discrepancies between our data and two previously established reference studies widely used in Spain: the Eurofit-Catalonia study, which collected data in 1984–85 but was published in 1998 [26], and the study by Marrodán Serrano et al., which collected data in 2006 and was published in 2009 [3].

As was the case in the study by Marrodán Serrano et al., the cross-sectional nature of our study limits the interpretation of the rate of change or strength acquisition over time. However, we consider that the sample size allows for the validation of the consistency of our results, potentially compensating for these limitations [3].

When comparing our results with the aforementioned references, differences are observed in the chronology of strength development and the absolute values of handgrip strength. These differences may be influenced by historical and/or sociological changes (such as the increase in sedentary activity at the expense of physical activity or the impact of the COVID-19 pandemic), as the samples do not differ significantly in terms of methodology or population characteristics.

**Figure 3:**
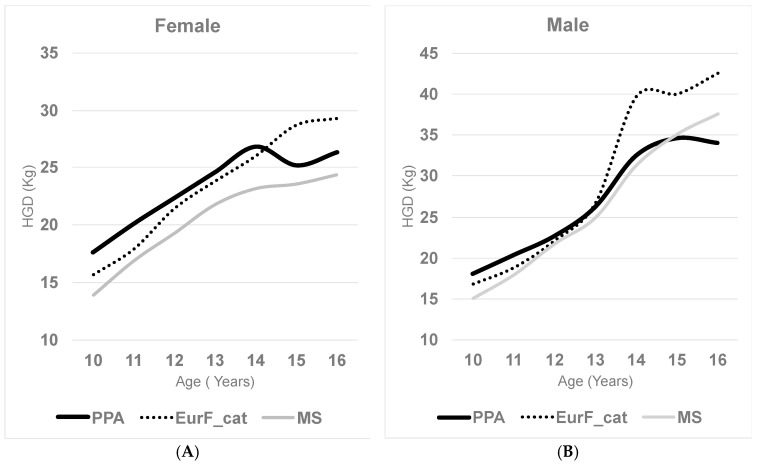
Comparative handgrip strength values: (**A**) female; (**B**) male. PPA: PESCA-PASOS-ASOMAD; EurF_cat: Eurofit; MS: Marrodán Serrano et al.

**Figure 4:**
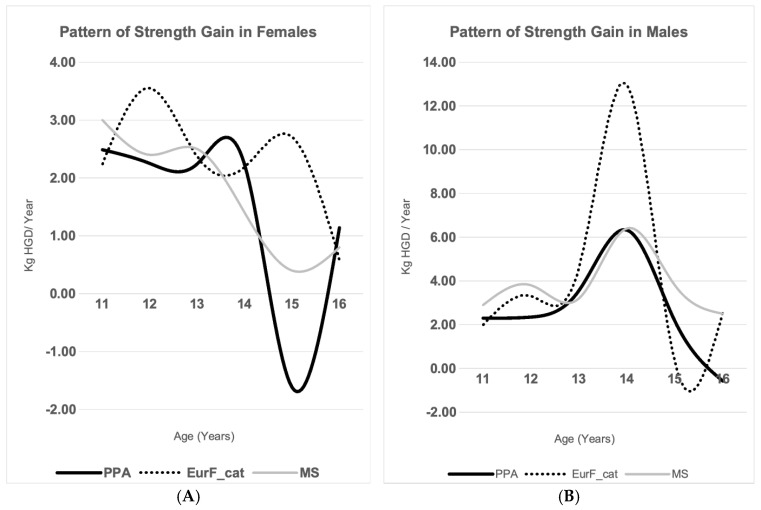
Comparative annual handgrip strength gain: (**A**) female; (**B**) male. PPA: PESCA-PASOS-ASOMAD; EurF_cat: Eurofit; MS: Marrodán Serrano et al.

Figure 3 and Figure 4 illustrate the different trajectories of strength development in both sexes across the three studies, while Table 4 presents the demographic characteristics of each sample. In general, the Eurofit-Catalonia study tends to show higher values and greater fluctuations in the rate of strength gain, particularly in males. In contrast, the study by M. Serrano et al. displays more stable and moderate increases [3], whereas our study appears to reflect an intermediate pattern. Although the primary aim of our comparison was to examine temporal trends within the Spanish population, international data also support similar findings. A recent systematic review identified modest but consistent declines in handgrip strength among children and adolescents in several countries over recent decades [12].

Notably, our results highlight a pronounced slowdown in the rate of strength gain (kg/year) in females between the ages of 14 and 15, where it even declines in absolute terms (−1.62 kg). Additionally, a reduction in the intensity of the male pubertal strength peak at age 14 is observed. This latter finding resulted in the 16-year-old males in our sample (the most recent cohort) being the weakest among the three study populations. Similarly, the percentile curves presented in Appendix A show an absolute decline in handgrip strength across most percentiles, occurring chronologically earlier in females (at ages 14–15) compared to males (ages 15–16).

Regarding the correlation with anthropometric variables, our data confirm the strong relationship previously reported by M. Serrano et al. [3] between handgrip strength and the subject’s weight, height, and age, as also described in other studies [18]. When introducing age and height as control variables, weight continues to show a significant positive correlation with handgrip strength, particularly in females.

It is well established that lean mass is likely the variable most strongly associated with handgrip dynamometry [3]; however, body composition data were not included in this study. Investigating this relationship could be a future research direction for our work.

When adjusting handgrip strength for body weight (kg HGD/kg body weight), we obtain a variable with lower allometric dependence [27]. Significant sex differences are also observed in this case, with this coefficient being higher in males (0.482 vs. 0.457), suggesting that, in addition to having greater muscle mass, males achieve higher efficiency in generating strength per unit of body weight. We also consider it of interest for future studies to further explore this coefficient as a more sensitive marker of handgrip strength. As a limitation, this study did not include information on pubertal development, which can influence handgrip strength independently of chronological age and sex. Future research should consider biological maturation to better understand strength variability during adolescence. Although pubertal development is recorded in the PESCA study through Tanner staging, this information is not available in the PASOS and ASOMAD datasets, which prevents its inclusion in the present pooled analysis.

Although the main goal of this study was to update the national handgrip strength reference values and analyze their temporal evolution, international studies also suggest similar trends. For instance, Laurson et al. reported that muscular strength and fitness in U.S. youth may have declined over time [28], and Dooley et al. described global trends pointing in the same direction [12]. Additional recent studies from Poland and the United States have also proposed updated normative references, further emphasizing the influence of regional, anthropometric, and ethnic factors on strength development [29,30].

Although the sample was large and diverse, the geographic concentration in the Madrid region may limit the generalizability of the findings to other populations. Socioeconomic and environmental factors not directly assessed in this study may also influence strength development and should be considered in future research. It is worth noting that previous national reference studies used for comparison also shared similar limitations regarding geographical scope.

Future analyses using body composition data from the PESCA protocol, including lean and fat mass estimations via bioimpedance, will allow us to further explore the relationship between handgrip strength and specific body compartments.

## 5. Conclusions

Our study provides new percentile curves and tables for handgrip strength in the Spanish pediatric population, including, for the first time, data from the age of 3 years.

We emphasize the importance of considering sexual dimorphism in the assessment of muscle development, particularly from puberty onwards, and propose the use of handgrip dynamometry as a diagnostic tool in pediatric consultations based on this new reference.

When compared with historical datasets, we observed that 16-year-old adolescent males in our sample exhibited lower strength than their counterparts from 40 and 16 years ago.

We consider it essential for future studies to investigate the factors contributing to this decline in muscle strength, identify the underlying causes by analyzing their relationships with child and adolescent health in Spain, and evaluate interventions aimed at improving physical fitness in this population.

## Figures and Tables

**Table 1:** Descriptive statistics of the sample, by sex. HGD: hand grip dynamometry. BMI: body mass index. *M*: mean; *SD*: standard deviation. Bold *p*-values indicate statistical significance (*p* < 0.05).

	Total					Female			Male			
	*n*	*Min*	*Max*	*M*	*SD*	*n*	*M*	*SD*	*n*	*M*	*SD*	*p*
**HGD (Kg)**	3281	2.00	55.00	16.98	7.59	1608	16.10	6.66	1673	17.81	8.30	**<0.001**
**Age (Years)**	3286	3.00	16.97	9.66	3.15	1612	9.59	3.11	1674	9.72	3.18	0.079
**Weight (Kg)**	3286	9.30	100.70	36.25	15.02	1612	35.43	14.11	1674	37.04	15.82	**0.031**
**Height (cm)**	3286	86.70	188.00	137.53	19.07	1612	136.58	18.15	1674	138.44	19.88	**0.022**
**BMI (kg/m^2^)**	3286	10.30	38.24	18.27	3.59	1612	18.16	3.51	1674	18.38	3.66	0.206
**Sport Time (h)**	1680	0.00	16.00	3.47	3.10	810	3.02	2.81	870	3.89	3.30	**<0.001**
**ScreenTime (h)**	1654	0.00	59.00	12.60	7.71	798	12.25	7.50	856	12.93	7.88	0.023
**HGD/Kg body weight**	3281	0.11	0.84	0.47	0.11	1608	0.46	0.10	1673	0.48	0.11	**<0.001**
	***n***	***%***	***IC 95%***			***%***	***IC 95%***		***%***	***IC 95%***		***p***
**Overweight (%)**	779	23.71	22.49	24.93		22.89	21.17	24.61	24.49	22.76	26.22	0.281
**Obesity (%)**	189	5.75	5.08	6.42		5.27	4.36	6.19	6.21	5.24	7.18	0.247

**Table 2:** Right-hand handgrip strength percentiles by sex for ages 3 to 16 years. M: mean; SD: standard deviation. Bold values in the ‘*p*’ column indicate statistical significance (*p* < 0.05).

*Female*																	
*Age*	*n*	*M*	*SD*	*p3*	*p10*	*p20*	*p25*	*p30*	*p40*	*p50*	*p60*	*p70*	*p75*	*p80*	*p90*	*p97*	*p*
**3**	69	5.03	1.81	2.00	3.00	3.50	4.00	4.00	4.00	5.00	6.00	6.00	6.00	6.50	7.00	9.00	0.370
**4**	78	6.74	1.88	4.00	4.00	5.00	5.38	6.00	6.00	7.00	7.00	8.00	8.00	8.00	9.00	11.26	**0.013**
**5**	88	8.55	1.85	5.00	6.00	7.00	7.00	8.00	8.00	8.75	9.00	10.00	10.00	10.00	11.00	12.00	**0.026**
**6**	85	10.24	2.54	4.58	7.00	9.00	9.00	9.00	9.40	10.00	11.00	11.10	12.00	12.00	14.00	15.00	0.067
**7**	114	12.65	2.78	6.90	10.00	10.00	11.00	11.00	12.00	13.00	13.00	14.00	14.00	15.00	16.00	18.00	0.439
**8**	248	13.49	2.73	8.84	10.00	11.00	11.50	12.00	12.50	13.25	14.00	15.00	15.00	16.00	17.00	19.00	**<0.001**
**9**	221	15.90	2.67	11.00	12.00	14.00	14.00	14.50	15.00	16.00	16.50	17.00	17.65	18.00	19.00	21.67	**0.002**
**10**	195	17.57	3.53	12.00	14.00	14.50	15.00	15.00	16.00	17.00	18.50	19.50	20.00	20.00	22.00	25.12	0.139
**11**	185	20.06	3.77	14.00	15.00	17.00	17.60	18.00	18.90	20.00	20.78	22.00	23.00	23.50	24.94	28.00	0.671
**12**	107	22.31	4.86	14.70	16.44	18.06	18.50	19.00	20.44	21.90	23.00	24.96	25.60	26.08	29.44	33.02	0.579
**13**	67	24.55	4.36	16.08	20.00	20.00	21.30	22.00	23.40	24.50	25.00	26.30	27.00	27.98	30.20	34.96	0.207
**14**	50	26.78	4.49	18.55	20.46	21.76	22.80	23.95	25.24	27.00	28.50	29.70	30.25	31.00	32.00	35.47	**<0.001**
**15**	61	25.16	5.12	15.00	19.10	20.64	21.00	22.06	23.38	24.50	25.76	27.70	28.50	29.76	32.50	37.07	**<0.001**
**16**	40	26.30	4.91	16.08	20.00	22.02	22.90	23.44	25.00	26.30	27.50	29.55	30.23	30.88	32.95	35.69	**<0.001**
**Total**	**1608**																
*Male*																	
*Age*	***n***	***M***	***SD***	***p3***	***p10***	***p20***	***p25***	***p30***	***p40***	***p50***	***p60***	***p70***	***p75***	***p80***	***p90***	***p97***	***p***
**3**	79	5.32	1.63	2.20	4.00	4.00	4.00	4.00	5.00	5.00	5.00	6.00	6.00	7.00	7.50	9.80	0.370
**4**	91	7.47	1.86	4.00	5.00	6.00	6.00	6.00	7.00	7.00	8.00	8.70	9.00	9.00	10.00	11.00	**0.013**
**5**	81	9.28	2.16	4.46	7.00	7.70	8.00	8.00	9.00	9.00	10.00	10.00	11.00	11.00	12.00	13.27	**0.026**
**6**	74	11.09	2.31	7.25	8.00	9.00	9.50	10.00	10.00	10.50	12.00	12.25	13.00	13.50	14.25	15.00	0.067
**7**	88	12.90	2.71	7.01	10.00	11.00	11.00	11.00	12.00	13.00	14.00	15.00	15.00	15.10	16.00	17.67	0.439
**8**	246	14.88	3.07	9.71	11.00	12.00	12.50	13.00	14.00	15.00	15.84	16.50	17.00	17.24	19.00	20.00	**<0.001**
**9**	233	16.81	3.16	11.00	13.00	14.00	14.50	15.00	16.00	17.00	17.50	18.46	19.00	19.50	21.00	22.49	**0.002**
**10**	209	18.00	3.30	12.00	14.00	15.40	16.00	16.00	17.00	18.00	19.00	20.00	20.00	20.50	22.00	25.85	0.139
**11**	185	20.30	4.17	13.29	15.34	16.92	17.00	18.00	19.00	20.00	21.00	22.00	23.00	23.42	25.50	29.00	0.671
**12**	136	22.65	5.00	14.29	16.56	18.32	19.00	20.00	21.00	22.30	24.00	25.00	25.23	26.24	30.00	32.45	0.579
**13**	89	26.21	7.19	14.14	17.10	20.00	21.40	22.50	23.90	25.00	28.00	29.10	30.00	31.40	35.40	41.70	0.207
**14**	54	32.54	6.72	22.26	24.00	26.00	27.38	28.30	31.00	32.20	33.00	34.95	36.25	38.00	40.50	51.35	**<0.001**
**15**	81	34.70	6.60	23.73	25.58	28.00	30.00	31.00	33.46	35.00	35.44	38.04	38.75	39.64	42.40	50.54	**<0.001**
**16**	27	34.12	6.53	24.00	26.78	29.28	30.00	30.80	32.00	32.00	33.72	35.66	36.70	39.48	46.06		**<0.001**
**Total**	**1673**																

**Table 3:** Spearman’s rho correlation coefficients between handgrip strength and anthropometric variables. *p* < 0.001 for all coefficients. * Control variable: age. ** Control variables: age and height.

	Total (3281)	Female (1608)	Male (1674)	Female *	Male *	Female **	Male **
**Age**	0.869	0.876	0.864				
**Weight**	0.862	0.871	0.855	0.470	0.462	0.320	0.318
**Height**	0.892	0.896	0.889	0.422	0.402		
**BMI**	0.602	0.619	0.587	0.335	0.280	0.283	0.205

**Table 4:** Demographic characteristics of the three series. PPA: PESCA-PASOS-ASOMAD; EurF_cat: Eurofit; MS: Marrodán Serrano et al.

Study	Female	Male	Total	Data Date	Age	Sample
**EurF_cat**	872	952	1824	1984–1985	10 to 18	Cataluña
**MS**	949	1176	2125	2006	6 to 18	Madrid
**PPA**	1608	1673	3281	2018–2022	3 to 16	Madrid

**Figure A1:**
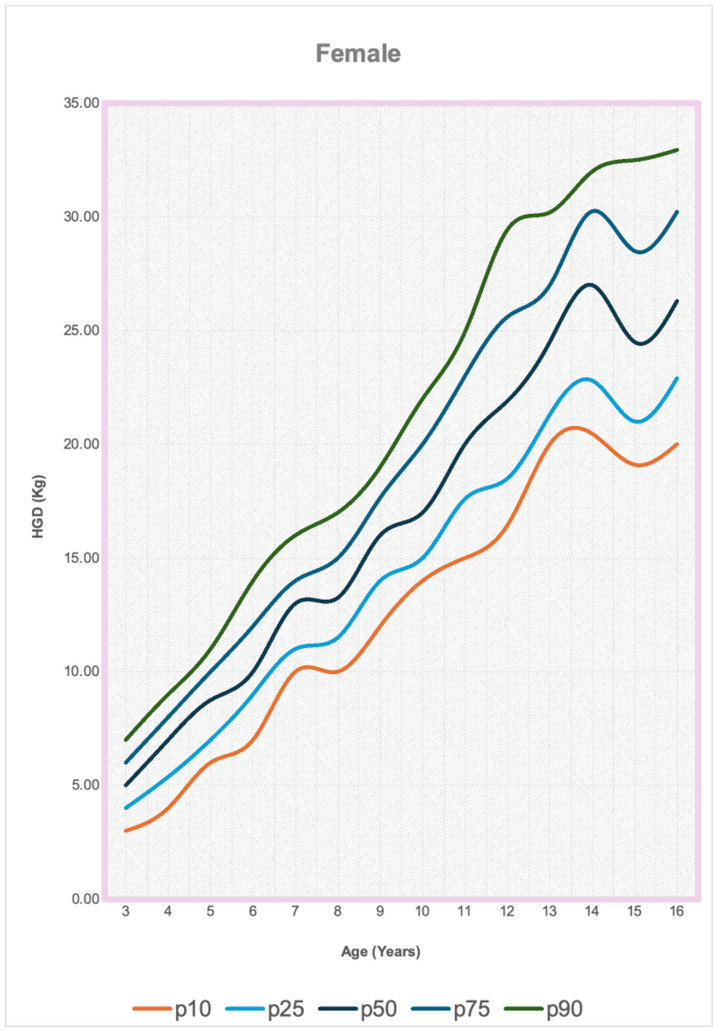

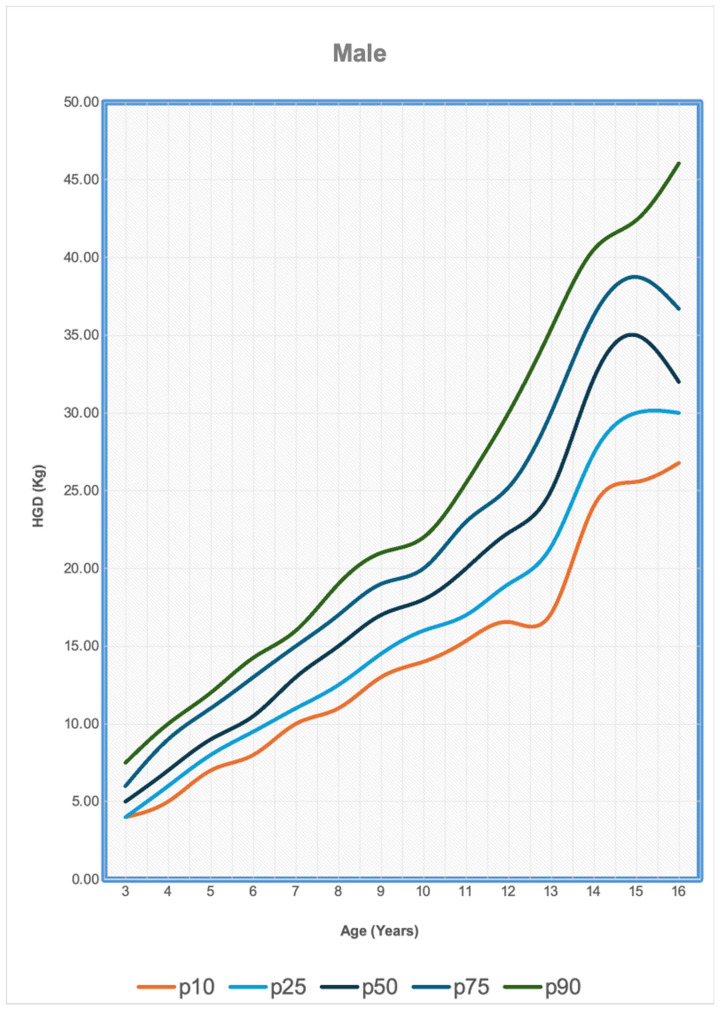
Percentile curves for right-hand handgrip strength by sex for ages 3 to 16 years.

## Data Availability

The data presented in this study are available on request from the corresponding author. The data are not publicly available due to privacy and ethical restrictions, as they include sensitive information derived from medical records and clinical examinations.
